# Fish Fillet Analogue Using Formulation Based on Mushroom (*Pleurotus ostreatus*) and Enzymatic Treatment: Texture, Sensory, Aromatic Profile and Physicochemical Characterization

**DOI:** 10.3390/foods13152358

**Published:** 2024-07-26

**Authors:** Nayara Thalita Ferreira Silva, Andreia Reis Venancio, Emerson Tokuda Martos, Ana Clara Gomes Oliveira, Ana Alice Andrade Oliveira, Yhan da Silva Mutz, Cleiton Antonio Nunes, Olga Lucía Mondragón-Bernal, José Guilherme Lembi Ferreira Alves

**Affiliations:** 1 Postgraduate Program in Food Engineering, Department of Food Science, Federal University of Lavras, P.O. Box 3037, Lavras 37203-202, Brazil; nayara_4@hotmail.com; 2 Department of Nutrition, Federal University of Lavras, P.O. Box 3037, Lavras 37203-202, Brazil; andreia.venancio@estudante.ufla.br; 3 Department of Food Science, Federal University of Lavras, P.O. Box 3037, Lavras 37203-202, Brazil; emerson.martos@ufla.br (E.T.M.); anaaliceoliveira@ufla.br (A.A.A.O.); yhan.mutz@ufla.br (Y.d.S.M.); cleiton.nunes@ufla.br (C.A.N.); olga@ufla.br (O.L.M.-B.); 4 Department of Chemistry, Federal University of Lavras, P.O. Box 3037, Lavras 37203-202, Brazil; ana.oliveira37@estudante.ufla.br

**Keywords:** hiratake *P. ostreatus*, transglutaminase, sensory analysis, texture profile analysis, e-nose, gas chromatography–mass spectrometry

## Abstract

The growing demand for alternative sources of non-animal proteins has stimulated research in this area. Mushrooms show potential in the innovation of plant-based food products. In this study, the aim was to develop prototype fish fillets analogues from *Pleurotus ostreatus* mushrooms applying enzymatic treatment (β-glucanase and transglutaminase-TG). A Plackett–Burman 20 experimental design was used to optimize forty variables. Oat flour (OF) exerted a positive effect on the hardness and gumminess texture parameters but a negative effect on cohesiveness and resilience. Soy protein isolate (SPI) exhibited a positive effect on elasticity, gumminess and chewiness, while acacia gum had a negative effect on elasticity, cohesiveness and resilience. After sensory analysis the assay with 1% cassava starch, 5% OF, 5% SPI, 0.1% transglutaminase (240 min/5 °C), 1% coconut oil, 1% soybean oil, 0.2% sodium tripolyphosphate, 0.6% β-glucanase (80 °C/10 min) and without β-glucanase inactivation was found to exhibit greater similarity to fish fillet. The classes hydrocarbons, alcohols and aldehydes are the predominant ones in aromatic profile analysis by chromatography and electronic nose. It is concluded that a mushroom-based analogue of fish fillet can be prepared using enzymatic treatment with TG.

## 1. Introduction

At a global level, contemporary consumers tend to be concerned with their well-being due to issues related to health, concerns about animals and awareness of the environment. Due to these factors, the number of vegetarians has increased, and a significant part of the population has reduced meat consumption; this habit is known as flexitarianism [1]. Several products are being investigated in which animal proteins are replaced with alternatives, including meat and fish analogues, as the demand for these products is increasing [2,3]. One raw material option for the formulation of meat-like products is edible mushrooms, which exhibit good nutritional, texture and flavor properties [4]. The potential of edible fungi and mycelium has been studied and recognized in the production of meat-like (included fish) foods due to their varied structures, appearance and composition, mainly of proteins, whose textures are similar to muscle and contain several bioactive compounds with beneficial effects on human health [5,6]. Among the bioactive compounds in mushrooms are triterpenes, phenolic compounds, sterols and (1,3)(1,6)-β-d-glucans, classified as dietary fibers. They are low-calorie products and classified as functional foods because they exhibit anticancer, antioxidant, antidiabetic and immunomodulatory effects [7]. The major indigestible polysaccharides present in fungi are chitin and β-d-glucans. They are composed of sugar units that are linked by β-glycosidic bonds. They are found in fungal fruiting bodies mainly in the vegetative and generative stages of ontogenesis with a structural function in the formation of cell walls in fungal cells and a physiological function of β-d-glucans when they form complexes with proteins as glycoproteins [8,9]. *P. ostreatus* glucans contain glucose as their main component, besides mannose and galactose [7]. In the fungal cell wall, proteins are distributed largely as glycoproteins, which are often crosslinked with polysaccharides, thereby providing protection [9]. The water holding capacity (WHC) of hydrated mushroom is a function of the integrity of the cell membrane as well as changes in the polymer structures and integrity of the cell wall, for example, after heat treatment. The WHC after heat treatment also depends on chitin, but mainly on the effect of pH change on the structure of proteins, being that the remaining WHC is caused by the osmotic binding of solutes and biopolymers present in the cell wall to water [9]. Fungal proteins also have good moisture retention capacity, good bioavailability, a high content of essential amino acids and glutamic acid, aspartic acid, or arginine and sulfur amino acids, which are precursors of animal protein flavor [5,6,10].

**Figure 1:**
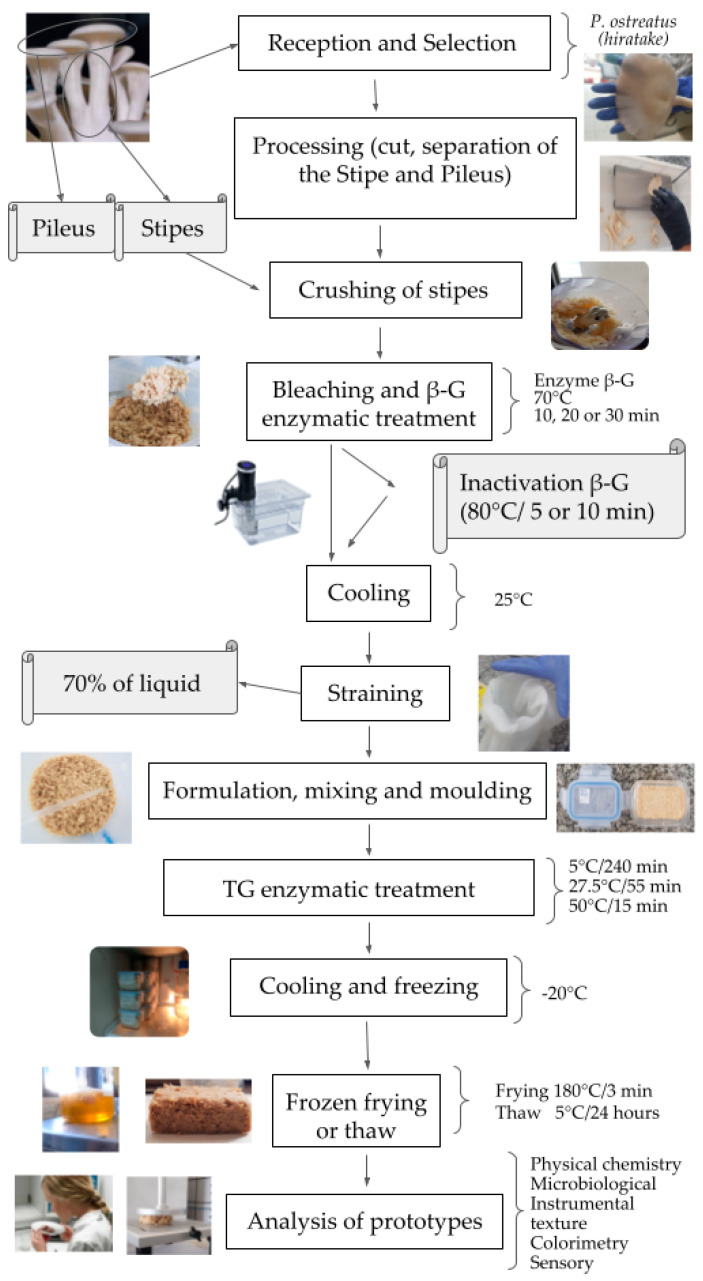
Processing flowchart for the fish fillet analogue.

The cultivation of *P. ostreatus*, also known as oyster or button mushroom, generates many mushrooms that do not meet the standards for commercialization in natura (because of size, shape and adult stage of maturation), causing significant losses for the producers [11], but can be used as raw material for the preparation of other food products. The white *P. ostreatus,* cultivated in Brazil, are popularly known as “Hiratake” when these mushrooms are harvested in a more developed state or “Shimeji” when harvested in the early stage [12]. These definitions will be adopted throughout the article for all mushrooms used in this study. The morphology of *P. ostreatus* mushrooms can be divided into two parts (pileus or cap and stipe), with the stem- or stalk-like feature supporting the pileus (Figure 1).

During the production of analogues, there is the challenge and concern of achieving textures, protein contents and sensory acceptance similar to the standard of animal origin. Heat treatments such as bleaching and pasteurization cause textural changes in mushrooms, such as increased force for puncturing or reduced force for shear and firmer texture [13,14]. Two of the technological processes to produce meat analogues are (1) the mixture of proteins and hydrocolloids [2] and (2) enzymatic crosslinking between food biopolymers, such as proteins of non-animal source [15]. In the first, fibers that biomimic muscle are obtained by mixing proteins with hydrocolloids that precipitate with multivalent cations. Various combinations of proteins, hydrocolloids, and cations can be used, such as soy protein and alginate [2]. In the second, proteins can be restructured through enzymatic crosslinking with the prior release and exposure of the protein [15]. This bioavailability of proteins and glycoproteins from mushroom cell walls for crosslinking could be achieved through enzymatic treatment with glucanase enzymes [16] and by solubilization through the addition of alkaline substances such as polyphosphates [17]. β-glucanase enzymes are responsible for the breakdown of β-glucan into low molecular weight moieties; this enzyme also enhances the solubility, bioactivity and functionality of hydrolyzed β-glucan. After depolymerization by enzymatic treatment, the structure of β-glucan changed from a semi-flexible chain to an extended random coil [16]. Some of the uses of food-grade phosphates include maintaining the structure and hydration of meat, poultry and seafood products (muscle foods) and use as a protein dispersant. Phosphates and sodium chloride allow for unbound proteins to form a sol (a colloid or an aggregate of very fine particles dispersed in a fluid), forming a viscous or sticky liquid [18] when mixed and to form a gel when heated and cooled. These processes improve binding and reduce moisture loss after cooking, increasing the juiciness of cooked products. Tripolyphosphates (for example, in fish products) solubilize the surface myofibrillar protein, providing stickiness and adhesion between fish fillets [17].

Among potential enzymes for protein crosslinking are transglutaminases (TGs) [17]. TGs are classified as EC2.3.2.13, being protein–glutamine γ-glutamyltransferase [19]. They belong to the group of acyltransferases that catalyze acyl transfer reactions between a γ-carboxyamine group of a peptide- or protein-bound glutamyl residue and a primary amino group of various substrates, including the ε-amino group of lysine or lysyl residues in proteins, resulting in polymerization or amine incorporation causing crosslinking or crosslinking by covalent crosslinks [17].

The objective of the work was to develop a process for preparing a fish fillet analogue based on *Pleurotus ostreatus* mushrooms, added with vegetable proteins, oils and gums and using an enzymatic method with β-glucanase and transglutaminase (TG). Furthermore, the analogues were characterized physicochemically and sensorially and their aromatic profile was determined.

## 2. Materials and Methods

### 2.1. Raw Materials

*P. ostreatus* mushrooms (Cogumelos do Japinha—Bom Sucesso/Brazil) were used as the main ingredient, with harvesting points of “Shimeji” and “Hiratake”. They were stored under refrigeration (4 °C) for up to 24 h after harvest by the producer until the experiments were performed; soy protein isolate (SPI) 90% (C2 Alimentos—Andradas/Brazil); whole oat flour—OF (Dr. Oetker, Porto Alegre, Brazil); glutamine—Gt (Generics Labs, São Paulo/Brazil); acacia gum—AG (arabica) (Nova Fórmula, Diadema/Brazil); cassava starch—CS (São Lourenço/Brazil); transglutaminase enzyme—TG (New Ingredients^®^, Bochum/Germany); β-glucanase enzyme—β-G (Prodooze B, Mogi das Cruzes/Brazil); monosodium glutamate—MG (Ajinomoto^®^, São Paulo/Brazil); Coconut oil—CO (copra, Sumaré/Brazil); soybean oil—SO (Soya^®^, Ponta grossa/Brazil); sodium tripolyphosphate—ST (Synth^®^, Diadema/Brazil).

### 2.2. Preparation of Fish Fillet-like Analogues and Experimental Design

To prepare the analogues, a *Plackett–Burman* (BP) experimental design was performed with 20 trials and 3 central points to find the best analogue prototypes of fish fillet [20]. The relationship of coded and real values of the independent variables of the Plackett–Burman experimental design 20 is provided in Table 1. The independent variables were the concentration of β-glucanase (β-G) (% *w*/*w*), action time of β-glucanase (min), concentration of the enzyme transglutaminase (TG) (% *w*/*w*), temperature/time binomial (min/°C) for TG, soy protein isolate (SPI) (% *w*/*w*), oat flour (OF) (% *w*/*w*), glutamine (Gt) (% *w*/*w*), monosodium glutamate (MG) (% *w*/*w*), acacia gum (AG) (% *w*/*w*), cassava starch (CS) (% *w*/*w*), coconut oil (CO) (% *w*/*w*), soybean oil (SO) (% *w*/*w*), sodium tripolyphosphate (ST) (% *w*/*w*) and time of β-glucanase inactivation (min). This enzyme was added to evaluate its effect on increasing the accessibility of mushroom proteins to react with transglutaminase and other plant proteins, since mushrooms are rich in β-d-glucans that surround the protein layer. The dependent variables were rheological parameters, such as hardness (N), elasticity (mm), cohesiveness (rate), gumminess (N), chewiness (N/mm), resilience (rate), CIELAB color parameters, water activity (a_w_) and pH. The coded matrix for the Plackett and Burman experimental design (PB20) containing the formulation and process conditions to elaborate the mushroom-based fish fillet analogue prototypes is detailed in Supplementary Materials. Statistical analyses were performed with Statistica 8.0 software (StatSoft Inc., Tulsa/OK, USA) [21].

The methodology used to prepare and assemble the formulations was adapted from [22] and is shown in Figure 1. The first stage involved receiving and cleaning the *P. ostreatus* mushrooms, which were cleaned with a brush, and the pileus were separated from the stipes. Next, the stems were ground using a Walita mixer model RI1363 and stored in 100 g hermetically sealed plastic bags; the enzyme β-glucanase (β-G) was added according to the *Plackett and Burman* experimental design (PB20) for each assay. The air was removed from the plastic bags, which were closed and taken to the thermostatic bath. Bleaching and enzymatic treatment with β-G were performed simultaneously at an optimal temperature of action (β-G 70 °C [23]), and the times were varied (10, 20 or 30 min) according to the PB20. After thermal treatment, the β-G assays were placed in an ice bath at 80 °C for 5 or 10 min [24], and all packages were placed in an ice bath until the temperature reached 25 °C.

Subsequently, the liquid exudate from the thermal treatment process was drained [25]. Thus, masses of treated and cooled stems were placed in polyester filters, and manual pressing was performed until 30% of the initial mass was obtained.

To perform the formulation of each test, 100 g per sample was used as a calculation basis. The powder ingredients were weighed and mixed as follows: TG, SPI, OF, Gt, MG, AG, CS and ST. SO and/or CO were added to the drained stem, and homogenization was performed for 3 min with a mixer. Subsequently, the mixture of powdered ingredients was added, and the mass was homogenized for 5 min. Transglutaminase was added to each formulation and the masses were molded in refractory containers that were kept in BOD at the temperature/time binomial according to PB20. After the enzyme action time, the analogues were cooled and frozen. The frozen samples for sensory analysis were fried in a standardized manner by immersion in soybean oil at 180 °C for 1.5 min on each side, enough time to reach 70 °C at the geometric center and forming a golden crust. The samples for other analyses such as texture, color, water activity, microbiological analyses and aromatic compounds were thawed at 5 °C for 24 h.

### 2.3. Analytic Methods

#### 2.3.1. Proximate Composition

The proximate composition of commercial-size (Shimeji) and noncommercial-size (Hiratake) *P. ostreatus* mushrooms and of the fractions of the mushrooms, divided into pileus and stipe, were determined. The analysis methods were adapted from the *Association of Official Analytical Chemicals* [26] for determination of moisture by oven drying (AOAC 925.09, 2005), protein by the Kjeldahl method (AOAC 920.87, 2005) with conversion factor 4.38, total lipids per Soxhlet extraction (AOAC 920.39, 2005), crude fiber (AOAC 991.43, 2005), ash by muffle incineration (AOAC 942.05, 2005) and glucose fraction (total carbohydrates), adding the numbers corresponding to the percentages of the five preceding determinations and subtracting 100. The analyses were performed in triplicate, and the values were expressed on an integral basis, also called a wet basis.

#### 2.3.2. Instrumental Texture Profile Analysis (TPA)

Texture analysis was carried out using a TA.XT plus Texture Analyzer (Stable Micro Systems Ltd., Godalming, UK), equipped with a 5 kg load cell. The parameters used were similar to those from [27]. A cylindrical probe P/75 mm in diameter was used, the samples were cut into cubes with an edge of 2.0 cm, and were compressed twice to 50% of their original thickness at a pre-test speed of 1 mm/s. The test and post-test were carried out at a speed of 2 mm/s between compression cycles, using the probe at an initial height of 30 mm. Firmness was obtained by analyzing the force curve as a function of time. The firmness variable, expressed in Newton (N), was recorded and from it the parameters of hardness, elasticity, cohesiveness, gumminess, chewiness and resilience were calculated using the equipment software. Five repetitions of the analyses were performed for each assay.

#### 2.3.3. Color Measurement

For color analysis, a Konica Minolta CM-5 colorimeter (Minolta, Osaka, Japan) was used. After calibration with a standard (white plate), the samples were placed on a transparent plate and analysis was carried out in triplicate. The results were expressed according to the CIE LAB system with reference to the illuminant D65 and a visual angle of 10°. The following parameters were determined: L*, a* and b*, where L* defines the lightness (L* = 0—black and L* = 100—white) and a* and b* are values indicating chromaticity (−a* green and +a* red; −b* blue and +b* yellow) [28].

#### 2.3.4. Sensory Analysis

The sensory analysis was performed after approval by the Committee of Ethics in Research (COEP) with humans in UFLA (Ethical approval No.: 5,552,956 on 29 July 2022). Microbiological analysis for *E. coli* was performed following AOAC methodology 992.30 [29] using the ColiComplete^®^ test kit from Merck (Barueri, Brazil). The tubes were examined under long-wave ultraviolet light (366 nm). Fluorescent tubes indicate a positive result for *E. coli*. All samples served in the sensory test tested negative for *E. coli*.

The sensory analysis was performed with 108 untrained volunteer tasters who were of both sexes, over 18 years old, attending the Federal University of Lavras and consumers of fish. Sensory evaluation was performed in individual booths at the Sensory Analysis Laboratory of UFLA with methodologies adapted from [30]. Each taster received 3 samples coded with 3 random numbers, monadic presentation, along with the evaluation form. The previously selected PB20 frozen samples were placed in a frying pan with soybean oil at 180 °C and fried on each side for 1.5 min. Soybean oil was chosen because it is cheaper and more consumed in Brazil. Then, the samples were removed, placed on absorbent paper sheets, cut into 2 cm cubes and served at a temperature between 40–45 °C.

The participants were given sensory evaluation sheets that contained five 9 cm unstructured hedonic scales to affectively test the appearance, aroma, flavor, texture and overall impression; then, the participants ranked the level by which the prototypes resembled fried fish in the evaluated aspects. The results obtained in the sensory analysis were subjected to analysis of variance (ANOVA), and the means were compared by Tukey’s test at the 5% significance level using Statistica 8.0 software [21].

#### 2.3.5. Aromatic Profile

The aromatic profiles of the three best fish fillet analogues of PB20 and raw *P. ostreatus* mushroom stipes were determined using HS-SPME-GC-MS and e-nose techniques. The fried analogues were prepared as described in item 2.3.

##### Headspace Solid-Phase Microextraction Combined with Gas Chromatography–Mass Spectrometry (HS-SPME-GC-MS)

The methodology for HS-SPME-GC-MS was adapted from [31]. Approximately 5 g of samples of raw mushroom strains of *P. ostreatus* and fried fish fillet analogues were crushed into pieces no more than 5 mm wide and placed in vials with a septum. Volatile organic compounds (VOCs) were headspace extracted using solid phase microextraction (SPME). The fiber used for extraction was SPME ASSY 50/30 PVB//CAR/DPMS SF 1 cm SUPELCO brand (Cajamar, Brazil). The extracted compounds were separated and identified using a gas chromatograph coupled to a GC-MS QP2010 Plus mass spectrometer (Shimadzu, Japan), equipped with an automatic liquid and gas injector AOC-5000 (Shimadzu, Japan) and an SLBTM column (5% phenyl-95% dimethylsiloxane) with dimensions of 30 m × 0.25 mm × 0.25 μm. The injector temperature was maintained at 230 °C and operated in non-splitless mode. The carrier gas used was He with a purity of 99.999%, with a flow rate of 0.8mL/min. The oven temperature was programmed to maintain the temperature at 40 °C for 5 min initially, and then increase to 230 °C at a heating rate of 5 °C min^−1^, maintaining this temperature for 10 min. The mass spectrometer used is equipped with an electron impact ionization source (70 eV) using scan mode (45 to 500 Da), and the interface and ion source temperatures were 230 °C and 200 °C, respectively. The VOCs were identified by comparing the mass spectra of the sample with those in the Mass Spectral Database Wiley 8-GCMSQP2010 Plus Shimadzu_GCMS solution ver. 2.5 and the experimentally obtained retention indices with those reported in the literature [32]. Experimental retention indices were obtained by injecting a homologous series of alkanes (C8–C20).

##### E-Nose Analysis

The in-house electronic nose built was based on 12 metal oxide semiconductors sensors (MQ-2, MQ-3, MQ-4, MQ-5, MQ-6, MQ-7, MQ-8, MQ-9, MQ-135, MQ-136, MQ-137 and MQ-138) arranged in an analysis chamber (350 mL) through which the sample vapors circulated. Each sensor outputs a voltage, which is proportional to the presence of vapors it is sensible to. These outputs were read as an analogic signal every 500 ms using an Arduino Mega and the SVisual Monitor (version 1.21, available at https://github.com/Tyill/SVisual (accessed on 1 June 2024). The methodology was adapted using the same binomials of time and temperature for the purpose of comparing results obtained by e-nose and gas chromatography. Around 5.0 g of the samples was put into a closed glass bottle (250 mL) and kept at 40 °C for 30 min in an incubator with the aim of forming headspace with volatile compounds. After this time, the samples were transferred and circulated through the sensor chamber for using an air circulation pump at a flow rate of 1 mL/min, for 3 min. A 5 min purge of atmospheric air was performed between each sample reading to allow for the recovery of the baseline signals. After signal acquisition, the average value of the 3 min read for each sensor was obtained, resulting in a fingerprint of 12 sensor signals for each sample (adapted from [33,34]).

## 3. Results

### 3.1. Proximate Composition

Table 2 shows the values obtained from the proximate analyses performed with whole mushrooms and mushrooms fractionated into the stipe and pileus of *P. ostreatus* in the young commercial (Shimeji) and adult noncommercial standard (Hiratake) maturation stages.

No statistically significant difference was observed for moisture between the contents of whole shimeji mushrooms (92.64%) and those of whole Hiratake mushrooms (92.66%) and between the moisture content of the pileus (93.01%) and stipe (92.17%) fractions of the Hiratake mushrooms. Moisture above 90.65% for *P. ostreatus* was found by [35]. Approximate values (91.34% and 91.80%) were found in other studies [25,36], respectively. A 77.13% moisture content in tilapia fillets was determined by [37], thus lower than in mushrooms.

The ether extract (lipid) levels found in this study were low, as expected for mushrooms, and ranged from 0.03% (Hiratake pileus) to 0.11% (whole Shimeji); these values were lower than those of tilapia fillet. No significant difference was observed between the contents of whole Hiratake (0.05%) and Shimeji (0.11%) and of the stem (0.08%). An ether extract content of 0.15% for the same mushroom species was found by [35] and higher values (0.36%) were found by [25]. It was found lipid levels that ranged from 0.10% (stage 1 of growth) to 0.12% (stage 2) in the *Agaricus bisporus* mushroom stipe and from 0.10% (stage 4) to 0.20% (stage 1) in the pileus [36]. Low-fat foods are important for healthy, calorie-restricted diets. A lipid content of 2.60% was found in tilapia, a result higher than that found in the mushrooms in this study [37].

The protein content of the mushrooms ranged from 1.2% to 2.1%, and no statistically significant difference was observed at 5% between whole shimeji (2.07%), hiratake mushrooms (1.54%) and hiratake pileus (1.78%). In the *P. ostreatus* mushrooms evaluated by [35], the protein content was 3.40%. Similar values were reported by [25,38]. The protein content of *P. ostreatus* mushrooms may be between 0.8 and 2.4% in wet weight basis [39], a range that encompasses the value obtained in this study. For *A. bisporus* mushrooms, ref. [40] concluded that the crude protein ranged from 3.83% to 5.56% in the stipe and from 4.37% to 6.18% in the pileus. The highest levels of crude protein were found in the stipe and pileus of mushrooms harvested at growth stage 1 (smaller size). The variability in the protein content of mushrooms results from the influence of several factors, such as the composition of the cultivation substrate, maturation stage of the mushroom, climatic variations and management technique [40]. The protein content of tilapia fillets was determined by [37] in 19.4%. As the mushrooms used in this study generated lower values than the references found for fish fillet, the protein content in the formulation of the mushroom-based fish analogues was adjusted to approximate the amount provided by a freshwater fish fillet, such as tilapia, by adding vegetable protein sources, such as soy protein isolate.

**Table 2:** Values obtained for the proximate and physicochemical composition of *P. ostreatus* Shimeji and Hiratake.

Analyses	Shimeji	Hiratake			*P. ostreatus*	Tilapia Fillet
	Integer	Integer	Pileus	Stipe	Ref. [35]	Ref. [37]
Moisture (%) *	92.64 ± 0.11 ^a^	92.66 ± 0.13 ^a^	93.01 ± 0.91 ^a^	92.17 ± 0.39 ^a^	90.65	77.13
Ethereal extract (%) *	0.11 ± 0.02 ^b^	0.05 ± 0.01 ^ab^	0.03 ± 0.01 ^a^	0.08 ± 0.04 ^ab^	0.15	2.6
Crude protein (%) *	2.07 ± 0.29 ^a^	1.54 ± 0.07 ^ab^	1.78 ± 0.29 ^a^	1.18 ± 0.13 ^b^	3.4	19.36
Gross fiber (%) *	0.25 ± 0.16 ^c^	1.69 ± 0.22 ^ab^	1.09 ± 0.41 ^a^	1.76 ± 0.14 ^b^	3.06 **	-
Ash (%) *	0.67 ± 0.02 ^c^	0.46 ± 0.01 ^a^	0.57 ± 0.04 ^b^	0.43 ± 0.02 ^a^	0.76	1.09
Carbohydrates (%) *	4.26 ± 0.22 ^a^	3.83 ± 0.51 ^a^	3.52 ± 0.82 ^a^	4.37 ± 0.67 ^a^	1.98	-
pH	6.59 ± 0.07 ^a^	6.44 ± 0.20 ^a^	6.44 ± 0.20 ^a^	6.42 ± 0.14 ^a^	-	-
Acidity (% m/m) ***	0.18 ± 0.01 ^a^	0.14 ± 0.04 ^a^	0.17 ± 0.06 ^a^	0.14 ± 0.01 ^a^	-	-
Aw	0.98 ± 0.00 ^a^	0.98 ± 0.01 ^a^	0.99 ± 0.00 ^a^	0.99 ± 0.00 ^a^	-	0.983

Legend: * Values in g·100 g^−1^; ** dietary fiber; *** values in g of citric acid/100 g of mushroom. Different letters on the same line differ from each other statistically (5% significance).

Total fiber and ash contents of 0.25% and 0.67% were found for whole shimeji mushrooms and 1.69% and 0.46% for hiratake, respectively, with a significant difference (*p* < 0.05). The results are similar to those obtained by [41], who analyzed *P. ostreatus* mushrooms and obtained 0.86% for fibers and 0.61% for ash on a wet basis. Authors of [35] found 3.06% of total fibers and 0.76% of ash, with higher contents, which can be explained by the culture medium and substrate used. According to [42], the variation in fiber content may be mainly related to genetic factors, which determine the amount and type of saccharides present in fungal cell walls. According to [37], the ash content of tilapia is 1.09% and *P. ostreatus* can be classified as foods with low ash content.

The glucose fraction content of the mushrooms ranged from 3.52% (Hiratake pileus) to 4.37% (Hiratake stipe), but there was no significant difference. Lower values (1.98%) were found by [37], and higher values (6.69%) were found by [25]. The carbohydrates found in mushrooms are mainly polysaccharides that constitute the cell wall. Cell walls contain a mixture of fibrillary components that include chitin and the polysaccharides β-D-glucans and mannans. These components are nondigestible carbohydrates that are resistant to human enzymes and provide a source of dietary fiber [39,42].

Based on these results, the mass of the mushroom-based product must be enriched with protein and lipid sources to elaborate a prototype that mimics fish, especially tilapia species.

Higher pH values were found in this study for shimeji (6.59) than for the whole Hiratake, pileus and stipe (6.44, 6.44 and 6.42, respectively), but there was no significant difference (*p* < 0.05). For *A. bisporus* mushrooms, pH values of 6.6 and 6.8 were found by [43,44], respectively. Raw tilapia fillets generally exhibit pH around 6.6 and 7.0 [45], values close to those of the mushroom.

Acidity in foods influences their flavor, color, microbial stability and general quality [46]. The total titratable acidity results ranged between 0.18 g/100 g (Shimeji) and 0.14 g/100 g (whole and stem hiratake), but there was no significant difference.

Regarding water activity (a_w_), the values for the whole shimeji and Hiratake mushrooms were high (0.98), with no significant difference (*p* < 0.05), agreeing with the value 0.994 found by [36] for fresh *P. ostreatus*. For raw tilapia, [37] found a_w_ of 0.983, which confirms the perishability of these foods.

As the mean values found for the proximate and physicochemical composition of the shimeji and hiratake mushrooms of *P. ostreatus* were similar, the Hiratake mushrooms were chosen to prepare the analogue because the commercial value of these mushrooms is lower.

### 3.2. Development of a Mushroom-Based Fish Fillet-like Analogue

The responses of Plackett–Burman design for the prototypes of the analogue of fried fish fillet and for the samples of fried tilapia were texture profile (hardness, elasticity, cohesiveness, gumminess, chewiness, resilience), instrumental color (CIELAB) and physicochemical parameters (a_w_ and pH) can be seen in Table 3.

Hardness is the force needed to compress food between the molar teeth. In this experiment, the hardness values ranged from 155 N (treatment 20) to 725 N (treatment 17), a value closer to that of the tilapia fillet (1507 N). The results showed that the hardness values of the analogues were lower than those of the fried tilapia fillet.

Elasticity is the distance that the sample recovers after the first compression (mm); that is, elasticity is defined as the ratio between the distance travelled around the second compression cycle A2 and the distance travelled in the area of the first compression cycle A1. For this parameter, the results obtained for the analogues were below the fried tilapia fillet, ranging from 0.23 mm (treatment 7) to 0.49 mm (treatment 5), the value closest to that of the fillet (0.57 mm). A high elasticity value indicates that the tested material is broken into large fragments during the first TPA compression. In contrast, a low elasticity value indicates that the material is destroyed into many small fragments. The elasticity value of a plant-based meat analogue [47] decreased with the content of isolated rice protein (IRP) in relation to soy protein isolate (SPI) and it was concluded that the gelling ability of SPI was better than that of IRP.

Cohesiveness is the ratio calculated as the ratio of the second compression area to the first compression area. Thus, the values obtained for the analogues in relation to this parameter ranged between 0.22 (treatment 13) and 0.38 (treatment 5), which was the last treatment above the cohesiveness of the fried tilapia (0.37). Authors of [47] observed that the cohesiveness of the analogues improved with the use of SIP compared to textured soy protein (TSP) because the analogues formed stronger protein–intermolecular protein interactions.

Gumminess is the force needed to disintegrate the mass of food until it is swallowed. For this parameter, all samples were below the tilapia fillet (560 N), ranging between 42 N (treatment 18) and 181 N (treatment 17). Chewiness is the energy needed to chew a solid food until swallowing, and the values ranged between 13 N/mm (treatment 18) and 58 N/mm (treatment 17), lower than the value of the tilapia fillet (318 N/mm).

Resilience measures the response of the mass after compression when the compressive force is interrupted and is determined by the work of shrinkage divided by the work of compression. This parameter was between 0.06 (treatments 7, 13 and 18) and 0.17 (treatment 5), with the latter being higher than the value of the tilapia fillet (0.11). In the studies performed by [48], increasing the frying ratio significantly increased hardness, gumminess and chewiness. Another aspect that can increase the hardness is the type of protein. According to [49], higher levels of pea protein isolates (up to 17%) increased the hardness and chewiness of meat analogues. These authors attributed the remarkable increase in structural strength to a more extensive network of protein crosslinks. A common feature of vegetable protein-based meat analogues is that the conformation of the protein/protein-based fibrous structures is tightened when subjected to some type of pressure, while juiciness and moisture retention decrease [50].

In the development of a hamburger analogue with *Flammulina velutipes* mushroom and TG [51], the presence of the mushroom increased the porosity of the hamburger microstructure and the TG addition significantly decreased the size of the holes, forming a uniform and compact microstructure. The enzyme decreased the amount of free amino acids and soluble proteins and induced the formation of new proteins with high molecular weights. The covalent crosslinking catalyzed by TG helped form a stronger gel matrix in the hamburger, leading to higher hardness, adhesiveness, chewiness, water-holding capacity and sensory acceptance.

The texture parameters, especially hardness, gumminess and chewiness, of the analogues were significantly lower than the parameters observed for the tilapia fillet. This result can be explained by the characteristics of the protein matrix in the fish fillet, the presence of fat or emulsions and the immobilization of water in the muscle tissue, which increased the hardness in the meat system [50,52]; these effects occur in the same manner in the analogue.

**Figure 2:**
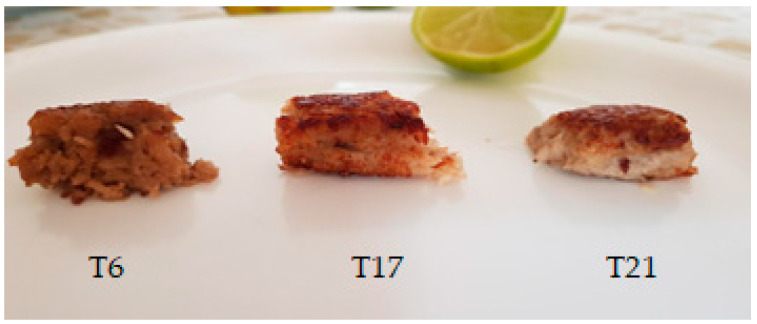
Samples from treatments 6, 17 and 21 (central point—CP) for sensory analysis.

When measuring food color, the L* a* b* color space is among the most used parameters due to the uniform distribution of colors and because it is perceptibly uniform, i.e., the Euclidean distance between two different colors approximately corresponds to the difference in color perceived by the human eye [53]. In this study, the L* values found for the analogue ranged from 37 (treatment 10) to 56 (treatment 19), and for the tilapia fillet, the value was 40. The higher the L* value is, the lighter the sample [29] (CIE, 1986); therefore, the lightness of the analogues was similar to that of the fish fillet. The results for the chromatic parameter a* ranged from 5 (treatment 12) to 10 (treatment 4), with 14 for the tilapia fillet. Positive values of a* demonstrate that the product tends to exhibit a reddish color [28]; thus, even below the tilapia fillet, the values were very close to the parameter. The results for chromatic b* ranged from 13 (treatment 17) to 24 (treatment 9), and the mean value for tilapia was 21. The positive b* plane indicates that the product exhibits yellowish tones [28]; therefore, the results also showed that the values are close to the standard of tilapia. Mazumder et al. (2023) developed an emulsified sausage based on mushrooms and chickpeas. They found values of approximately 88 (L*), 3 (a*) and 6 (b*) in the treatment with cooking and without beet-based dye. It is often difficult to imitate the color change that occurs during cooking in meats. Authors of [48] observed that increasing the proportion of frying significantly increased a* and b*. Generally, during the frying process, foods undergo physical and chemical changes, such as caramelization and the Maillard reaction. These changes significantly affect the color of fried foods [48]. Therefore, food-grade ingredients or dyes must be used, whether natural or artificial, that are heat stable during cooking or frying. The temperature and frying time can be adjusted to achieve color parameters closer to those of fried fish. Due to the hardness, gumminess and chewiness parameters of the prototypes in the 23 treatments, assays 6 and 17 and 21 (central point) (Figure 2) showed values close to those of the fried tilapia fillet and were chosen for sensory analysis.

#### 3.2.1. Plackett–Burman (PB20) Statistical Analysis

The significant effects (*p* < 0.1) of the 14 variables studied in PB20 on the 12 responses are shown in Table 4 and are highlighted in bold.

For hardness, it was observed that OF exhibited a significant positive effect; thus, within the study range (0–5%), the higher OF percentage added in the formulation, the greater the hardness of the fish fillet analogue. As the hardness of fried tilapia is 1507 N, the formulation could be enriched with more OF. Other ingredients, such as SPI, Gt, MG, AG, CS, CO, SO and ST showed no significant effect.

In elasticity, SPI and the temperature/time binomial for TG had a significant positive effect, while AG had a negative effect. In analogues, elasticity is positively correlated with protein concentration [54], that is, if the protein concentration decreases, the average elasticity also decreases [55], but this result may be altered in gum presence. The increase in the SPI content and the temperature/time binomial for TG caused greater gelation in the analogue, increasing its elasticity.

Regarding cohesiveness, only oat flour (OF) and gum (AG) exerted significant and negative effects, showing that the increase in the content of these two ingredients decreases the interaction between the proteins of the analogue. Regarding gumminess, the variables that had significant positive effects were SPI and OF. On chewiness, only SPI had a significant positive effect. As the protein content increases, more proteins are available for crosslinking, resulting in a firmer structure [56] for chewiness, hardness and gumminess. In [55] it was observed that as the oat protein content increased in the mixture with pea protein for the extruded analogue, cohesiveness increased, justifying that the three-dimensional internal structure in analogues is maintained by hydrophobic interactions and stabilized by hydrogen bonds and disulfide. Regarding resilience of fried analogues, OF, AG and SO caused significant negative effects on. Resilience refers to how well a sample recovers from deformation in terms of speed and strength, while cohesiveness is a measure of the strength of the internal bonds within the matrix and the extent of deformation that can be withstood before rupture [57]. The addition of starchy ingredients decreases the resilience and cohesion of the analogues. Large values of cohesion and resilience show that the bonds that hold the matrix together are stronger, because the greater resilience may be related to the lower water retention capacity and greater internal crosslinking, creating a firm network that is not easily deformed. Low values of cohesiveness and resilience, such as those observed with high protein concentrations, result in weaker internal bonds and, therefore, greater degrees of deformation [58].

About color parameters, no variable caused significant effects on lightness (L*). CS and ST caused negative effects on a*, distancing it from the value of fried tilapia fillet. For parameter b*, the concentration of β-G had a negative effect, while the time of action of β-G and the binomial temperature/time of action of TG had positive effects. The contradictory effects of b-glucanase concentration (x1—negative effect) and the time of b-glucanase enzymatic treatment (x2—positive effect) on the b* parameter can be explained by the fact that the enzymatic treatment occurred simultaneously with bleaching. Therefore, the changes in the b* parameter probably occurred due to reactions and changes in the ingredients of the analogues due to greater exposure to the enzymatic treatment temperature of 70 °C and not due to the action of b-glucanase. Additionally, during the frying process, food undergoes physical and chemical changes, such as caramelization and the Maillard reaction. These changes significantly affect the color of fried foods [48].

For water activity (aw), the binomial temperature/time of TG action had a positive effect, while concentration of TG, SPI, Gt, AG and CS had negative effects, with increasing total solids content. For pH of fried analogue, the concentration of β-G had a negative effect, as did the temperature/time binomial.

The Aw is a relevant parameter for food shelf life, in combination with other factors (pH, salt, temperature, antioxidants and others) [59]. The activity of the treatment analogues varied between 0.69 (treatments 14 and 16) and 0.91 (treatment 20), with the average value for fried tilapia being 0.98. Therefore, the prototypes presented lower water activity than tilapia fillet. There is a positive correlation between moisture content and aw. Foods with aw between 0.700 and 0.850 are considered intermediate moisture foods and the growth of bacterial pathogens may have been inhibited in samples within this mentioned range [44]. Treatments with aw above 0.85 could be microbiologically unstable at room temperature. Another factor observed was the negative effect of TG enzyme on aw, that is, the increase in its concentration decreased the water activity of the analogue, possibly because the formation of a structure with stronger bonds retains less water.

The pH of the analogue varied between 6.5 and 7.1, values close to that of tilapia fillet (6.6). The addition of vegetable proteins such as SPI increased the pH of the vegan and vegetarian sausage formulation with *P. sapidus* in relation to the control sample [60].

#### 3.2.2. Sensory Analysis

The three treatments that presented the best texture and color results (6, 17 and 21) were sensorially evaluated (Figure 2), and the results are shown in Table 5, as well as the levels of their independent variables.

According to Table 5, higher scores for appearance, texture, flavor and overall impression were obtained for the samples from treatment 17, and acceptance was greater than that of treatments 6 and 21. In its formulation, treatment 17 was the only treatment that did not contain acacia gum (AG) and had higher contents of coconut oil (CO), soybean oil (SO), sodium tripolyphosphate (ST) and oat flour (OF) and a lower content of soy protein isolate (SPI). Regarding texture, oat flour may have contributed to a more fibrous texture and firmness, which was more pleasing to the tasters.

Texture is among the most important attributes that indicates quality in fish and fish products [61], and the analogue must exhibit a structure that resembles the myotomes of fish musculature. Regarding aroma, no significant difference was observed between the three samples. This may be related to the strong odor produced by SPI and frying. The main unpleasant odors of SPI under normal conditions and without lipoxygenase originate from hexanal and nonanal [62]. The flavor was the result that obtained the lowest scores by the panelists among all attributes. This occurred because frying in soybean oil can produce desirable or undesirable flavor compounds that can affect the final products [63]. The oil content in fried foods depends on the balance between moisture drainage and oil suction driven by porosity food for the outer layer [64].

The migration of water can lead to changes in the microstructure of food and the formation of porous channels [65], causing food to absorb more oil. Frying may have negatively affected the analogues and masked nondominant aromas of other ingredients in the formulation. The highest overall impression score was obtained for treatment 17, which may be related to a more balanced formulation between the ingredients compared to the other two treatments. However, the average score is still low, indicating that the tasters neither liked nor disliked the sample. In [22], sensory analysis on their ground beef analogue created with *P. sajorcajur* mushroom was presented. The results showed that the general acceptability of the base formulation is “like slightly”. This is not surprising, as this is a new type of food, and many consumers reject food during the initial consumption. This lower acceptance may be linked to food neophobia, a condition in which consumers doubt and resist new foods, leading to a tendency to regularly consume the same types of foods [66]. The differences between fish analogues in terms of sensory quality and fried fish are due to the unique morphological and sensory characteristics that each raw material has regarding its composition. The results are important for the development and optimization of a process and formulation of an analogue to fish fillet.

### 3.3. Aromatic Profile

The evaluation of the aromatic profile was carried out by comparing HS-SPME-GC-MS analysis, electronic nose (e-nose) and sensory analysis. In Supplementary Materials, the results of the aromatic profile from HS-SPME-GC-MS of treatments 6, 17 and 21 of the fish fillet analogue prototypes are presented and compared with the main raw material (fresh Hiratake stipe) and with tilapia fried in soybean oil reported by [32].

As can be seen in Supplementary Materials, a total of 34, 47 and 28 volatile compounds were identified for samples from treatments 6, 17 and 21, respectively, through HS-SPME-GC-MS analysis and odor descriptors were consulted in databases [67,68,69]. The classes hydrocarbons, alcohols and aldehydes are the predominant ones in aromatic profile analysis by chromatography. This can also be observed in the analysis carried out with e-nose, in which the highest conductance recorded in the graph was for alcohols and hydrocarbons. In contrast, in the hiratake stipe mushroom, 21 organic components have been identified, the majority being alcohols. These results are in line with those observed in [70], in which six functional groups (alkene, alkane, alcohol, aldehyde, ketone and other organic compounds) as the main volatile ones, with alcohols, aldehydes and ketones dominating among the volatile components, were identified from *P. ostreatus* mushrooms.

Components found in common between analogues from this study and tilapia fried in soybean oil [32] include 2,3-Butanedione (creamy), Phenylmethanal (almond), Hexanal (Vegetative), Octanal (orangepeel), Octanal (Malty), 2-Amylfuran (fruity) and 1-octen-3-ol (mushroom). Other components found in tilapia fried [32] and which were not detected in the analogues were 2-ethylpyrazine (roasted), 2,6-dimethylpyrazine (roasted), 2,3-Dimethyl-5-ethylpyrazine (burnt popcorn), Trimethylamine (fishy), amylalcohol (balsam), Methylpyrazine(nutty), acetonealcohol(burnt), 2,3-dimethylpyrazine (nutty), dimethyltrisulfide (meaty), 2-ethyl-6-methylpyrazine (roasted potato), nonanal (fatty), 2,3,5-trimethylpyrazine (nutty), trans-2-octenal (cucumberoily), 2,3,5,6-tetramethylpyrazine(nutty), (E)-2-nonenal(cucumber), 2-propyl-Pyridine(roasted), gamma-butyrolactone (fatty), furan-2-ylmethanol (bready),2-hexylthiophene (floral), (E,E)-2,4-Nonadienal (fatty), 2-undecenal (fruity), (E,E)-2,4-Decadienal (fatty), (R)-(-)-pantolactone (cotton candy). Most belong to the pyrazine family.

Pyrazines are important components in the aroma of fried fish [32], and are not present in the analogues prepared in this study. It is interesting to look for plant-based ingredients that contain pyrazine precursors such as the free amino acids asparagine, serine, hydroxyamino acids, as well as sugars such as glucose, fructose or sucrose [71], or hydroxamic acid [72]. According to [71], protein-bound amino acids are important precursors of pyrazines, and they suggest that threonine is an amino acid linked to proteins that undergoes greater pyrolytic degradation, having a direct relationship with their formation. The absence of pyrazines can be attributed to the low content of free hydroxyl amino acids, not producing detectable amounts of pyrazines by chromatographic method.

Pyrazines can be generated directly by the Maillard reaction, by Strecker degradation or by pyrolysis of hydroxy amino acids. In the “Maillard’’ reaction, carbohydrate degradation products are the main source of carbon for the formation of pyrazines, while amino acids function as sources of nitrogen for these compounds [71]. Free amino acids represent a small proportion of protein nitrogen; however, they are extremely important aroma precursors, with a marked participation in the quality of the final product. Pyrazine derivatives vary from both a quantitative and qualitative point of view, depending on the amino acid/glucose system used. Pyrazine derivatives are obtained in systems containing hydroxy amino acids and sucrose, or containing serine/glucose, or serine/fructose, the latter being a generator of greater quantities of pyrazines. Therefore, the quantity and variety of pyrazines formed depend on the ratio between the amount of amino acid and sugar in the reaction system. In Strecker degradation, pyrazines are formed by a-diketones as carbon sources and nitrogen from amino acids. A series of pyrazines were identified from reactions of alanine with a-diketones (e.g., 2,3-butanedione, present in treatment 17 and which gives a creamy and buttery odor). In reaction systems containing threonine, serine and sucrose [73], it was demonstrated that alkyl pyrazines are generated in greater quantities in the serine/threonine system than in the serine/threonine/sucrose system from the pyrolysis of hydroxy amino acids, undergoing decarboxylation and dimerization.

The volatile compounds found in fish snacks made from different surimi, cassava starch and baking powder using e-nose [74] agreed with some of the analogues in this study: ethanol (pungent, strong, sweet, weak) and propane (odorless). Levels of ammonia were detected as possible precursors of pyrazines [74].

Some natural sources of threonine among proteins of plant origin are beans, corn, soy derivatives, wheat, pea and other sees [75]. Serine contains an α-amino group, a carboxyl group that in pH∼7.4 is a polar amino acid. Some vegetable sources of serine are soybeans, nuts (especially peanuts, almonds and walnuts), chickpeas and lentils [76].

Trimethylamine is an important compound in the aromatic profile of fish which, according to [32], provides a fishy odor, but was not detected in any of the treatments studied. Trimethylamine is an organic compound with the formula N(CH_3_)_3_, it is a nitrogenous base and a product of the decomposition of plants and animals but is responsible for the unpleasant odor associated with decomposing fish, some infections and even bad breath.

In this study, 17 alcohols were found, with 7-Octen-4-ol or 1-Octen-5-ol being the most abundant in the mushroom stipes, and which curiously provide a characteristic aroma of soy, followed by ethanol and 1-Butanol, 3-methyl providing aromas of liqueurs such as wine, whiskey and malt, respectively. 3-Octanol, which is described in the literature as a mushroom aroma and was not found in the stipes, but was detected in the analogues, mainly in the treatment 21. These compounds come mainly from the decomposition of fatty acids by fatty acid precursor enzymes, unsaturated fatty acids and contribute significantly to the aroma of fungi [34] and the analogues were formulated with coconut oil and soy oil in the case of T17 and T21. Interestingly, the compound 1-octen-3-ol or amyl vinyl carbinol that provides a mushroom taste was not present in the *P. ostreatus* mushroom stipe. There are some common components found in the *P. ostreatus* mushroom [77] and in this study, such as nonano, which was exclusively found in the stipe (Supplementary Materials). In addition, the following substances were identified: octane, ethanol and 3-octanone, the latter being the component found in the greatest quantity in mushrooms. The disulfide compound was also detected in reasonable quantities in the research. In this study, benzaldehyde and hexanal are present in the analogue samples, as well as in the fried fish [32] and in mycelium of *P. ostreatus* [34]; however, they were not detected in the hiratake stipes, showing that these aromatic compounds probably being present in the mycelium, in the pileus and not in the stipes of the mature mushroom. Other ingredients of the formulations may have been responsible for the presence of these aldehydes, such as SPI, OF and others

**Figure 3:**
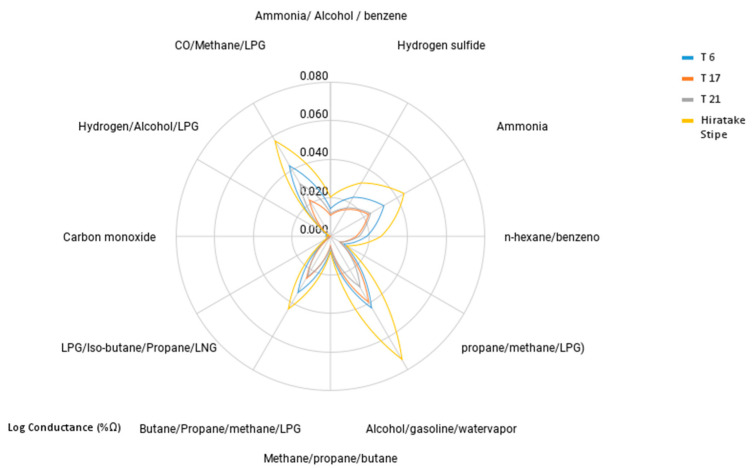
Radar fingerprint plot for volatile compounds identified in T6, T17 and T21 samples of fish fillet analogues and in stipe of *Pleurotus ostreatus* mushrooms by e-nose analysis.

In Figure 3, using a radar graph, there are the log values of the electrical conductance (S) of the functional groups detected by the sensors using the e-nose for each of the samples analyzed (Treatments 6, 7, 21 and *P. ostreatus* strains). All treatments showed the same aromatic behavior, and in treatment 6, higher conductance values were obtained, which means that there is a higher content of components analyzed in each sensor. The functional groups with the highest conductances in the analyzed samples were alcohol (0.043–0.078 log conductance), CO/Methane/LPG (0.042–0.047 log conductance), ammonia (0.032 to 0.044 log conductance) and butane/propane/methane/LPG (0.034 at 0.044 log conductance). The stipes of *P. ostreatus* (hiratake) showed the highest percentages, followed by treatment 6 (lowest sensory acceptance), while T21 and T17 (with better sensory acceptance) showed similar results for ammonia and butane/propane/methane/LPG. This behavior is expected since the aromatic components of the stipes are diluted in the analogue formulations when mixed with other ingredients. Treatment 6 contained a higher content of SPI (soy protein isolate), which could be related to this aromatic profile with lower acceptance.

Negative notes in the sensory analysis may be related to the high concentration of alkanes, which give off unpleasant odors similar to those of gasoline. Among the hydrocarbons, pentane was the most predominant in the four samples, contributing with aromas of petroleum derivatives. Hydrocarbons are often associated with unpleasant odors. A good part of the off-flavors can be attributed to the mushroom, influenced by several factors such as the harvest, the specific batch of mushroom or the bleaching process, among others. Among the treatments, T17 was the one that presented the best sensory evaluation and was also the one that showed the lowest hydrocarbon content in the e-nose, a fact that demonstrates a possible correlation between sensorial acceptance and hydrocarbon content. In this study, bleaching was carried out in sous vide (vacuum), a process that concentrates the aromas of the food and favors reactions for the formation of new aromas, a fact that was counterproductive for the mushrooms flavor. A possible improvement in sensorial acceptance could occur conducting bleaching by direct immersion in water to eliminate some of these undesirable aromatic compounds.

Another component that can be highlighted in the classification of alcohols is ethanol, which was present in significant quantities in the four samples. In treatment 6, the largest amount of this component was detected compared to the others. Treatment 17 was the one with the highest amount of aldehydes, including the compound phenylmethanal, which has pleasant odors of fruits and leaves, which contributed to this treatment obtaining more positive and acceptable sensory notes.

## 4. Conclusions

The use of *P. ostreatus mushrooms,* especially its stem fraction, is promising in the preparation of fish fillet analogues; however, due to their low protein and lipid levels, other ingredients should be added to the formulation. The additives that showed the greatest effects on the texture parameters of the analogues were oat flour (hardness and gumminess) and soy protein isolate (elasticity, gumminess and chewiness). Regarding color, the variables evaluated did not significantly affect the lightness of the samples (L*), while cassava starch and sodium tripolyphosphate exerted significant effects on the a* parameter, decreasing its chromaticity. Within the studied range, the concentration of TG significantly altered the a_w_ of the analogues but not the texture parameters. The results show that the formulation and process must be optimized to increase parameters, such as hardness, gumminess and chewiness, to more closely resemble a fish fillet, as well as improve appearance, flavor and aroma. The sensory analysis showed that treatment 17, composed of TG (0.1%), SPI (5%), OF (5%), MG (2%), CS (1%), CO (1%), SO (1%), ST (0.2%), and β-G (0.6%), led to a greater acceptance for appearance, texture, flavor and overall impression. The classes hydrocarbons, alcohols and aldehydes are the predominant ones in aromatic profile analysis by chromatography e-nose. Negative notes in the sensory analysis may be related to the high concentration of alkanes. However, neither pyrazines nor trimethylamines were detected, which cause characteristic fishy odors, and it is therefore important to add plant-based precursor sources of these aromas in future optimizations.

## Figures and Tables

**Table 1:** Coded and real values used in Plackett–Burman experimental design.

Variables	Code	Real Values		
		−1	0	1
β-G concentration * (%)	x1	0	0.3	0.6
Time with β-G (min)	x2	10	20	30
Concentration TG ** (% *w*/*w*)	x3	0.1	0.55	1
TG temperature/time binomial (°C/min)	x4	5/240	27.5/55	50/15
SPI *** (% *w*/*w*)	x5	5	7.5	10
Oat flour—OF (% *w*/*w*)	x6	0	2.5	5
Glutamine—Gt (% *w*/*w*)	x7	0	1	2
Monosodium Glutamate—MG (% *w*/*w*)	x8	0	1	2
Acacia gum—AG (% *w*/*w*)	x9	0	5	10
Cassava starch—CS (% *w*/*w*)	x10	0	0.5	1
Coconut oil—CO (% *w*/*w*)	x11	0	0.5	1
Soybean oil—SO (% *w*/*w*)	x12	0	0.5	1
Sodium tripolyphosphate—ST (% *w*/*w*)	x13	0	0.1	0.2
β-G inactivation (min)	x14	0	5	10

* β-G: β-glucanase; ** TG: transglutaminase; *** SPI: soy protein isolate.

**Table 3:** Physical–chemical, texture and color parameters of the analogue prototypes of fried fish fillet and fried tilapia fillet for PB20.

Coded Matrix PB20															PB 20 Response Matrix—Fried Prototypes										
Treat.	x1	x2	x3	x4	x5	x6	x7	x8	x9	x10	x11	x12	x13	x14	Hard	Elas	Coe	Gum	Che	Res	L*	a*	b*	a_w_	pH
1	1	−1	1	1	−1	−1	−1	−1	1	−1	1	−1	1	1	234	0.32	0.27	64	21	0.1	43	7	17	0.9	6.5
2	1	1	−1	1	1	−1	−1	−1	−1	1	−1	1	−1	1	321	0.4	0.33	105	41	0.1	48	8	21	0.9	6.7
3	−1	1	1	−1	1	1	−1	−1	−1	−1	1	−1	1	−1	363	0.41	0.32	115	46	0.1	52	7	21	0.8	6.7
4	−1	−1	1	1	−1	1	1	−1	−1	−1	−1	1	−1	1	402	0.39	0.31	126	50	0.1	43	10	21	0.8	6.9
5	1	−1	−1	1	1	−1	1	1	−1	−1	−1	−1	1	−1	220	0.49	0.38	82	40	0.2	50	6	20	0.9	7
6	1	1	−1	−1	1	1	−1	1	1	−1	−1	−1	−1	1	629	0.35	0.26	164	58	0.1	45	7	19	0.8	6.5
7	1	1	1	−1	−1	1	1	−1	1	1	−1	−1	−1	−1	381	0.23	0.23	90	22	0.1	46	6	16	0.8	7.1
8	1	1	1	1	−1	−1	1	1	−1	1	1	−1	−1	−1	205	0.36	0.28	52	18	0.1	44	8	21	0.8	7.1
9	−1	1	1	1	1	−1	−1	1	1	−1	1	1	−1	−1	373	0.36	0.29	107	38	0.1	43	9	24	0.8	6.8
10	1	−1	1	1	1	1	−1	−1	1	1	−1	1	1	−1	372	0.29	0.25	93	28	0.1	37	7	14	0.7	6.6
11	−1	1	−1	1	1	1	1	−1	−1	1	1	−1	1	1	283	0.36	0.27	75	25	0.1	52	6	18	0.9	7
12	1	−1	1	−1	1	1	1	1	−1	−1	1	1	−1	1	403	0.33	0.26	105	36	0.1	48	5	15	0.8	7.1
13	−1	1	−1	1	−1	1	1	1	1	−1	−1	1	1	−1	559	0.27	0.22	124	33	0.1	50	8	20	0.9	6.8
14	−1	−1	1	−1	1	−1	1	1	1	1	−1	−1	1	1	291	0.32	0.31	91	30	0.1	43	6	15	0.7	7.1
15	−1	−1	−1	1	−1	1	−1	1	1	1	1	−1	−1	1	233	0.37	0.3	68	26	0.1	49	6	18	0.9	6.8
16	−1	−1	−1	−1	1	−1	1	−1	1	1	1	1	−1	−1	276	0.32	0.27	74	26	0.1	52	6	20	0.7	7.1
17	1	−1	−1	−1	−1	1	−1	1	−1	1	1	1	1	−1	725	0.31	0.25	181	58	0.1	42	7	13	0.9	6.9
18	1	1	−1	−1	−1	−1	1	−1	1	−1	1	1	1	1	165	0.3	0.26	42	13	0.1	49	6	17	0.8	6.8
19	−1	1	1	−1	−1	−1	−1	1	−1	1	−1	1	1	1	168	0.33	0.29	51	18	0.1	56	6	21	0.7	6.9
20	−1	−1	−1	−1	−1	−1	−1	−1	−1	−1	−1	−1	−1	−1	155	0.37	0.32	49	19	0.2	43	9	16	0.9	7.1
21	0	0	0	0	0	0	0	0	0	0	0	0	0	0	482	0.33	0.27	99	41	0.1	52	8	22	0.8	7.1
22	0	0	0	0	0	0	0	0	0	0	0	0	0	0	417	0.32	0.25	140	32	0.1	45	7	19	0.8	7
23	0	0	0	0	0	0	0	0	0	0	0	0	0	0	375	0.34	0.26	165	37	0.1	48	6	17	0.8	6.9
Tilapia															1507	0.57	0.37	560	318	0.1	40	14	21	1	6.6

Hard = hardness; Elas = elasticity; Coe = cohesiveness; Gum = gumminess; Che = chewiness; Res = resilience; L*, a* and b* = CIELAB color parameters; a_w_ = water activity.

**Table 4:** Effects of independent variables on texture responses, instrumental color and physicochemical parameters of fried fish fillet analogues.

	Hard		Elas		Coe		Gum		Che		Res		L*		a*		b*		a_w_		pH	
	Ef	*p*	Ef	*p*	Ef	*p*	Ef	*p*	Ef	*p*	Ef	*p*	Ef	*p*	Ef	*p*	Ef	*p*	Ef	*p*	Ef	*p*
Mean	**338**	**0.00**	**0.30**	**0.00**	**0.3**	**0.00**	**99**	**0.00**	**36**	**0.00**	**0.10**	**0.00**	**46.8**	**0.00**	**6.9**	**0.00**	**18.32**	**0.00**	**0.81**	**0.00**	**6.87**	**0.00**
Curv.	174	0.40	0.00	0.60	0.00	0.20	71	0.20	1	1.00	0.00	0.10	3.4	0.61	0.5	0.72	2.26	0.46	−0.01	0.82	0.28	0.09
x1	55	0.40	0.00	0.50	0.00	0.40	−1	1.00	−1	0.90	0.00	0.10	−3.4	0.19	−0.4	0.44	**−2.10**	**0.09**	0.01	0.58	**−0.11**	**0.08**
x2	14	0.80	0.00	0.40	0.00	0.20	19	0.30	5	0.40	0.00	0.30	3.5	0.17	0.1	0.78	**2.57**	**0.04**	0.01	0.80	−0.06	0.26
x3	−37	0.60	0.00	0.30	0.00	0.70	−3	0.90	−2	0.80	0.00	0.70	−2.5	0.32	0.3	0.57	0.47	0.67	**−0.06**	**0.02**	0.03	0.61
x4	−35	0.60	**0.03**	**0.08**	0.00	0.40	3	0.90	4	0.50	0.00	0.10	−1.7	0.49	0.8	0.14	**2.18**	**0.08**	**0.04**	**0.06**	**−0.11**	**0.07**
x5	30	0.60	**0.03**	**0.08**	0.00	0.20	**33**	**0.08**	**17**	**0.03**	0.00	0.40	0.4	0.86	−0.8	0.15	0.52	0.64	**−0.04**	**0.09**	−0.03	0.57
x6	**194**	**0.01**	0.00	0.20	**−0.03**	**0.04**	**38**	**0.05**	9	0.20	**−0.02**	**0.02**	−0.9	0.70	−0.2	0.62	−1.74	0.14	0.02	0.33	−0.07	0.19
x7	−39	0.60	0.00	0.40	0.00	0.50	−9	0.60	−4	0.60	0.00	0.30	1.7	0.47	−0.5	0.28	−0.15	0.89	**−0.04**	**0.10**	0.26	0.00
x8	85	0.20	0.00	0.60	0.00	0.90	9	0.60	3	0.60	0.00	0.70	0.5	0.83	−0.6	0.23	0.41	0.71	−0.01	0.77	0.06	0.27
x9	27	0.70	**−0.06**	**0.00**	**−0.03**	**0.04**	−8	0.60	−11	0.10	**−0.04**	**0.00**	−2.2	0.37	−0.4	0.37	−0.80	0.47	**−0.04**	**0.07**	−0.16	0.02
x10	−25	0.70	0.00	0.10	0.00	0.40	−16	0.40	−10	0.20	0.00	0.40	0.4	0.86	**−1.0**	**0.07**	−1.24	0.28	**−0.04**	**0.09**	0.12	0.06
x11	−24	0.70	0.00	1.00	0.00	0.40	−7	0.70	−1	0.90	0.00	0.30	1.1	0.63	−0.6	0.27	0.14	0.90	0.01	0.80	0.00	0.99
x12	77	0.30	0.00	0.10	0.00	0.20	−10	0.60	−8	0.30	**−0.02**	**0.05**	0.4	0.87	0.4	0.42	0.60	0.59	−0.02	0.29	−0.05	0.39
x13	0	1.00	0.00	0.70	0.00	0.80	7	0.70	4	0.60	0.00	0.40	1.2	0.60	**−0.9**	**0.09**	−1.56	0.18	0.01	0.55	−0.08	0.18
x14	−50	0.50	0.00	0.70	0.00	0.70	−18	0.30	−7	0.30	0.00	0.50	1.6	0.50	−0.4	0.40	−0.14	0.90	−0.01	0.58	−0.08	0.16

The values highlighted in bold mean statistically significant effects on responses (*p*-value < 0.1). Legends: Hard = hardness; Elas = elasticity; Coe = cohesiveness; Gum = gumminess; Che = chewiness; Res = resilience; L*, a* and b* = CIE color parameters [30]; aw = water activity; Curv. = curvature; X1 = β-glucanase concentration (%*w*/*w*); X2 = β-glucanase action time (min); X3 = transglutaminase (TG) concentration (%*w*/*w*); X4 = temperature/time binomial (min/°C) for TG; X5 = soy protein isolate—SPI (%*w*/*w*); X6 = oat flour—OF (%*w*/*w*); X7 = glutamine—Gt (%*w*/*w*); X8 = monosodium glutamate—MG (%*w*/*w*); X9 = acacia gum—AG(%*w*/*w*); X10 = cassava starch—CS (%*w*/*w*); X11 = coconut oil—CO (%*w*/*w*); X12 = soybean oil—SO (%*w*/*w*); X13 = sodium tripolyphosphate—ST (%*w*/*w*); X14 = β-glucanase inactivation time (min); Ef = effect; Er = pure error; *p* = *p*-value (<0.10).

**Table 5:** Results of affective sensory analysis.

Treatment	Appearance	Texture	Aroma	Flavor	Global Impression
6	3.1 ± 2.3 ^a^	3.4 ± 2.3 ^a^	3.1 ± 2.4 ^a^	2.0 ± 2.0 ^a^	2.9 ± 2.2 ^a^
17	5.5 ± 2.3 ^c^	4.5 ± 2.6 ^b^	3.5 ± 2.3 ^a^	3.0 ± 2.4 ^b^	4.0 ± 2.6 ^b^
21 (CP)	4.3 ± 2.7 ^b^	3.3 ± 2.4 ^a^	3.3 ± 2.3 ^a^	2.0 ± 1.9 ^a^	3.0 ± 2.4 ^a^

Different letters on the same column differ from each other statistically (5% significance). (CP): central point treatment. Treatment 6: β-G concentration 0.6%; β-G action time: 30 min; TG concentration: 0.1%; temperature/time for TG action: 5 °C/240 min; SPI: 10%; OF: 5%; Gt: 0%; MG: 2%; AG: 10%; CS: 0%; CO: 0%; SO: 0%; ST: 0%; β-G inactivation time at 80 °C: 10 min. Treatment 17: β-G concentration: 0.6%; β-G action time: 10 min; TG concentration: 0.1%; temperature/time for TG action: 5 °C/240 min; SPI: 5%; OF: 5%; Gt: 0%; MG: 2%; AG: 0%; CS: 1%; CO: 1%; SO: 1%; ST: 0.2%; β-G inactivation time at 80 °C: 0 min. Treatment 21(CP): β-G concentration 0.3%; β-G action time: 20 min; TG concentration: 0.55%; temperature/time for TG action: 27.5 °C/55 min; SPI: 7.5%; OF: 2.5%; Gt: 1%; MG: 1%; AG: 5%; CS: 0.5%; CO: 0.5%; SO: 0.5%; ST: 0.1%; β-G inactivation time at 80 °C: 5 min.

## Data Availability

The original contributions presented in the study are included in the article/Supplementary Materials, further inquiries can be directed to the corresponding author.

## Supplementary Materials

The following supporting information can be downloaded at: https://www.mdpi.com/article/10.3390/foods13152358/s1, Table S1: Plackett & Burman experimental design with 20 trials (PB20) and 3 central points matrix; Table S2: Aroma-active compounds identified in T6, T17 and T21 samples of fish fillet analogues and in stipe of Pleurotus ostreatus Hiratake mushrooms by HS-SPME-GC-MS analysis [32,67,68,69].

## References

[1] Lupetti C., Casselli R. (2024). Olhar 360° Sobre o Consumidor Brasileiro e o Mercado Plant-Based 2023/2024.

[2] Lima M., Costa R., Rodrigues I., Lameiras J., Botelho G. (2022). A Narrative Review of Alternative Protein Sources: Highlights on Meat, Fish, Egg and Dairy Analogues. Foods.

[3] Meyer F., Hutmacher A., Lu B., Steiger N., Nyström L., Narciso J.O. (2023). Vegan shrimp alternative made with pink oyster and lion’s mane mushrooms: Nutritional profiles, presence of conjugated phenolic acids, and prototyping. J. Curr. Res. Food Sci..

[4] Beluhan S., Ranogajec A. (2011). Chemical composition and non-volatile components of Croatian wild edible mushrooms. Food Chem..

[5] Singh U., Tiwari P., Kelkar S., Kaul D., Tiwari A., Kapri M., Sharma S. (2023). Edible mushrooms: A sustainable novel ingredient for meat analogs. eFood.

[6] Łysakowska P., Sobota A., Wirkijowska A. (2023). Medicinal Mushrooms: Their Bioactive Components, Nutritional Value and Application in Functional Food Production—A Review. Molecules.

[7] Vetter J. (2023). The Mushroom Glucans: Molecules of High Biological and Medicinal Importance. Foods.

[8] Cerletti C., Esposito S., Iacoviello L. (2021). Edible Mushrooms and Beta-Glucans: Impact on Human Health. Nutrients.

[9] Paudel E.P., Boom R.M., Haaren E.V., Siccama J., Smanl R.G.M. (2016). Effects of cellular structure and cell wall components on water holding capacity of mushrooms. J. Food Eng..

[10] Bohrer B.M. (2019). An investigation of the formulation and nutritional composition of modern meat analogue products. Food Sci. Hum. Wellness.

[11] Lim S.H., Lee Y.H., Kang H.W. (2013). Efficient Recovery of Lignocellulolytic Enzymes of Spent Mushroom Compost from Oyster Mushrooms, *Pleurotus* spp., and Potential Use in Dye Decolorization. Mycobiology.

[12] Dias E.S. (2010). Mushroom cultivation in Brazil: Challenges and potential for growth. Ciênc. Agrotec..

[13] Zivanovic S., Buescher R. (2004). Changes in mushroom texture and cell wall composition affected by thermal processing. J. Food Sci..

[14] Zivanovic S., Buescher R., Kim S.K. (2003). Mushroom Texture, Cell Wall Composition, Color, and Ultrastructure as Affected by pH and Temperature. J. Food Sci..

[15] Buchert J., Cura D.E., Ma H., Gasparetti C., Monogioudi E., Faccio G., Mattinen M., Boer H., Partanen R., Selinheimo E. (2010). Crosslinking Food Proteins for Improved Functionality. Annu. Rev. Food Sci. Technol..

[16] Kaur R., Sharma M., Ji D., Xu M., Agyei D. (2020). Structural Features, Modification, and Functionalities of Beta-Glucan. Fibers.

[17] Lampila L.E. (2013). Applications and functions of food-grade phosphates. Ann. N. Y. Acad. Sci..

[18] Innocenzi P. (2020). Understanding sol–gel transition through a picture. A short tutorial. J. Sol-Gel Sci. Technol..

[19] Duarte L., Matte C.R., Bizarro C.V., Ayub M.A.Z. (2020). Transglutaminases: Part I—Origins, sources, and biotechnological characteristics. World J. Microbiol. Biotechnol..

[20] Rodrigues M.I., Iemma A.F. (2014). Experimental Design and Process Optimization.

[21] StatSoft Inc (2008). STATISTICA (Data Analysis Software System), Version 8.0..

[22] Mazumder M.A.R., Sukchot S., Phonphimai P., Ketnawa S., Chaijan M., Grossmann L., Rawdkuen S. (2023). Mushroom–Legume-Based Minced Meat: Physico-Chemical and Sensory Properties. Foods.

[23] Fukuda K., Hiraga M., Asakuma S., Arai I., Sekikawa M., Urashima T. (2008). Purification and characterization of a novel exo-beta-1,3-1,6-glucanase from the fruiting body of the edible mushroom Enoki (*Flammulina velutipes*). Biosci. Biotechnol. Biochem..

[24] Buckow R., Heinz V., Knorr D. (2012). Effect of High Hydrostatic Pressure-Temperature Combinations on the Activity of β-Glucanase from Barley Malt. J. Inst. Brew..

[25] Manzi P., Aguzzi A., Pizzoferrato L. (2001). Nutritional Value of Mushrooms Widely Consumed in Italy. Food Chem..

[26] Association of Official Agricultural Chemists—AOAC (2005). Official Methods of Analysis of AOAC International.

[27] Bernardo Y.A., Rosario D.K.A., Monteiro M.L.G., Mano S.B., Delgado I.F., Conte C.A. (2022). Texture Profile Analysis: How Parameter Settings Affect the Instrumental Texture Characteristics of Fish Fillets Stored Under Refrigeration?. Food Anal. Methods.

[28] Commission Internationale de L’éclairage—CIE (1986). Colorimetry.

[29] Latimer G.W. (2023). AOAC Official Method 992.30. Confirmed Total Coliform and *Escherichia coli* in All Foods: Substrate Supporting Disc Method (ColiComplete^®^). Official Methods of Analysis of AOAC International.

[30] Mondragón-Bernal O.L., Maugeri F.F., Alves J.G.L.F., Rodrigues M.I. (2012). Synbiotic Soy Beverages: Principles and Sensory Attributes. Handbook of Plant-Based Fermented Foods and Beverages.

[31] Li B., Kimatu B.M., Li C., Pei F., Hu Q., Zhao L. (2017). Analysis of volatile compounds in *L. edodes* blanched by hot water and microwave. Int. J. Food Sci. Technol..

[32] Liu M., Zhao X., Zhao M., Liu X., Pang Y., Zhang M. (2022). Characterization of the Key Aroma Constituents in Fried Tilapia through the Sensorics Concept. Foods.

[33] Mutz Y.S., Nunes C.A. Use of an electronic nose based on oxide sensors metal (MOS) for analyzing specialty coffees with different roasting points. Proceedings of the 15th SLACAN—Latin American Symposium on Food Science and Nutrition.

[34] Zhang Z., Zang M., Chen J., Zhang K., Wang S., Li D., Li X., Liu M., Pan X. (2024). Effect of the mycelium of oyster mushrooms on the physical and flavor properties of a plant-based beef analogue. LWT-Food Sci. Technol..

[35] Bach F., Helm C.V., Bellettini M.B., Maciel G.M., Haminiuk C.W. (2017). Edible mushrooms: A potential source of essential amino acids, glucans and minerals. Int. J. Food Sci..

[36] Pascual-Pineda L.A., Hernández-Marañon A., Castillo-Morales M., Uzárraga-Salazar R., Rascón-Díaz M.P., Flores-Andrade E. (2021). Effect of water activity on the stability of freeze-dried oyster mushroom (*Pleurotus ostreatus*) powder. Dry. Technol..

[37] Simões M.R., Ribeiro C.F.A., Ribeiro S.C.A., PARK K.J., MURR F.E.X. (2007). Composição físico-química, microbiológica e rendimento do filé de tilápia tailandesa (*Oreochromis niloticus*). Food Sci. Technol..

[38] Cohen N., Cohen J., Asatiani M.D., Varshney V.K., Yu H.T., Yang Y.C., Li Y.H., Mau J.L., Wasser S.P. (2014). Chemical composition and nutritional and medicinal value of fruit bodies and submerged cultured mycelia of culinary-medicinal higher Basidiomycetes mushrooms. Int. J. Med. Mushrooms.

[39] Cheung P.C.K. (2013). Mini-review on edible mushrooms as source of dietary fiber: Preparation and health benefits. Food Sci. Hum. Wellness.

[40] Raya M., Shalaby M., Hafez S., Hamouda A. (2014). Chemical composition and nutritional potential of some mushroom varieties cultivated in Egypt. J. Food Dairy Sci..

[41] Rop O., Mlcek J., Jurikova T. (2009). Beta-glucans in higher fungi and their health effects. Nutr. Rev..

[42] Mukhopadhyay R., Guha A.K. (2015). A comprehensive analysis of the nutritional quality of edible mushroom *Pleurotus sajor-caju* grown in deproteinized whey medium. LWT-Food Sci. Technol..

[43] Fidanza M., Sanford D., Beyer D., Aurentz D. (2010). Analysis of Fresh Mushroom Compost. HortTechnology.

[44] Boonsiriwit A., Xiao Y., Kathuria A., Lee Y.S. (2022). Effect of moisture-controlled packaging treatment with acid-modified expanded vermiculite-calcium chloride on the quality of fresh mushrooms (*Agaricus bisporus*) during low-temperature storage. J. Sci. Food Agric..

[45] Zhao Y., Yang S., Yang X., Li L., Hao S., Cen J., Zhang H. (2019). Effects of Ozonated Water Treatment on Physico-chemical, Microbiological and Sensory Characteristics Changes of Nile Tilapia (*Oreochromis niloticus*) Fillets during Storage in Ice. Ozone Sci. Eng..

[46] Tyl C., Sadler G.D., Nielsen S.S. (2017). pH and Titratable Acidity. Food Analysis-Food Science Text Series.

[47] Lee J.S., Oh H., Choi I., Yoon C.S., Han J. (2022). Physico-chemical characteristics of rice protein-based novel textured vegetable proteins as meat analogues produced by low-moisture extrusion cooking technology. LWT-Food Sci. Technol..

[48] Yang Y., Qiu W., Tao N., Jin Y., Feng Y., Jin Y. (2021). Effect of ratio of oil to sample on the quality of fried fish (*Pseudorasbora parva*). J. Food Process. Preserv..

[49] Yuliarti O., Kovis T.J.K., Yi N.J. (2021). Structuring the meat analogue by using plant-based derived composites. J. Food Eng..

[50] Sha L., Xiong Y.L. (2020). Plant protein-based alternatives of reconstructed meat: Science, technology, and challenges. Trends Food Sci. Technol..

[51] Zou Y., Yang C., Wang N., Zheng Q.-W., Ye Z.-W., Wei T., Zhong J.-R., Guo L.-Q., Lin J.-F. (2023). Development of *Flammulina velutipes*-based meat analogs with tunable physicochemical, structural, and sensory properties. Int. J. Food Eng..

[52] Ismail I., Hwang Y.H., Joo S.T. (2019). Interventions of two-stage thermal sous-vide cooking on the toughness of beef semitendinosus. Meat Sci..

[53] León K., Mery D., Pedreschi F., León J. (2006). Color measurement in L*a*b* units from RGB digital images. Food Res. Int..

[54] Wee M.S.M., Goh A.T., Stieger M., Forde C.G. (2018). Correlation of Instrumental Texture Properties from Textural Profile Analysis (TPA) with Eating Behaviours and Macronutrient Composition for a Wide Range of Solid Foods. Food Funct..

[55] Kaleda A., Talvistu K., Vaikma H., Tammik M.-L., Rosenvald S., Vilu R. (2021). Physicochemical, textural, and sensorial properties of fibrous meat analogs from oat-pea protein blends extruded at different moistures, temperatures, and screw speeds. Future Foods.

[56] Zhang J., Liu L., Zhu S., Wang Q. (2018). Texturisation Behaviour of Peanut–SoyBean/Wheat Protein Mixtures during High Moisture Extrusion Cooking. Int. J. Food Sci. Tech..

[57] Dobson S., Pensini E., Dupuis J., Yada R., Marangoni A. (2023). Synergistic interactions between pea protein isolate and rapid-swelling starch. Food Hydrocoll..

[58] Joshi M., Aldred P., Panozzo J., Kasapis S., Adhikari B. (2014). Rheological and microstructural characteristics of lentil starch–lentil protein composite pastes and gels. Food Hydrocoll..

[59] Li P., Wu G., Yang D., Zhang H., Qi X., Jin Q., Wang X. (2020). Effect of multistage process on the quality, water and oil distribution and microstructure of French fries. Food Res. Int..

[60] Stephan A., Ahlborn J., Zajul M., Zorn H. (2018). Edible mushroom mycelia of *Pleurotus sapidus* as novel protein sources in a vegan boiled sausage analog system: Functionality and sensory tests in comparison to commercial proteins and meat sausages. Eur. Food Res. Technol..

[61] Cheng X., Zhang M., Adhikari B. (2013). The inactivation kinetics of polyphenol oxidase in mushroom (*Agaricus bisporus*) during thermal and thermosonic treatments. Ultrason. Sonochem..

[62] Li X., Zhang W., Zeng X., Xi Y., Li Y., Hui B., Li J. (2023). Characterization of the Major Odor-Active Off-Flavor Compounds in Normal and Lipoxygenase-Lacking Soy Protein Isolates by Sensory-Directed Flavor Analysis. J. Agric. Food Chem..

[63] Choe E., Min D.B. (2007). Chemistry of deep-fat frying oils. J. Food Sci..

[64] Bouchon P., Hollins P., Pearson M., Pyle D.L., Tobin M.J. (2006). Oil Distribution in Fried Potatoes Monitored by Infrared Microspectroscopy. J. Food Sci..

[65] Zhang J., Liu Y., Fan L. (2020). Effect of Pore Characteristics on Oil Absorption Behavior during Frying of Potato Chips. Innov. Food Sci. Emerg. Technol..

[66] Muhammad R., Mohd A.I., Ahmad R., Hanan F. (2016). Psychological Factors on Food Neophobia among the Young Culinarian in Malaysia: Novel Food Preferences. Procedia Soc. Behav. Sci..

[67] FlavorNet: Database of Aroma Compounds. https://www.flavornet.org/flavornet.html.

[68] PubChem: Open Chemistry Database. https://pubchem.ncbi.nlm.nih.gov/.

[69] The Good Scents Company Good Scents Data: Aroma Chemical Information. https://www.thegoodscentscompany.com/data/rw1155991.html.

[70] Zhang Z.-M., Wu W.-W., Li G.-K. (2008). A GC—MS Study of the Volatile Organic Composition of Straw and Oyster Mushrooms During Maturity and its Relation to Antioxidant Activity. J. Chromatogr. Sci..

[71] De Maria C.A.B., Moreira R.F.A., Trugo L.C. (1999). Componentes voláteis do café torrado. Parte I: Compostos heterocíclicos. Quim. Nova.

[72] Akiyama M., Murakami K., Ikeda M., Iwatsuki K., Kokubo S., Wada A., Tokuno K., Onishi M., Iwabuchi H., Tanaka K. (2005). Characterization of Flavor Compounds Released During Grinding of Roasted Robusta Coffee Beans. Food Sci. Technol. Res..

[73] Baltes W., Bochmann G. (1987). Model reactions on roast aroma formation. 1. Reaction of serine and threonine with sucrose under the conditions on coffee roasting and identification of new coffee aroma compounds. J. Agric. Food Chem..

[74] Choi Y., Chae J., Kim S., Shin E.C., Choi G., Kim D., Cho S. (2023). Surimi for snacks: Physicochemical and sensory properties of fried fish snacks prepared from surimi of different fish species. Fish. Aquat. Sci..

[75] Pires C.V., Oliveira M.G.A., Rosa J.C., Costa N.M.B. (2006). Qualidade nutricional e escore químico de aminoácidos de diferentes fontes protéicas. Food Sci. Technol..

[76] Handzlik M.K., Metallo C.M. (2023). Sources and Sinks of Serine in Nutrition, Health, and Disease. Annu. Rev. Nutr..

[77] Radványi D. (2022). Smelling the difference: Separation of healthy and infected button mushrooms via microbial volatile organic compounds. Heliyon.

