# Supplementary material for: Fish Fillet Analogue Using Formulation Based on Mushroom (Pleurotus ostreatus) and Enzymatic Treatment: Texture, Sensory, Aromatic Profile and Physicochemical Characterization
Source: Foods. 2024 Jul 26;13(15):2358. doi: 10.3390/foods13152358 (PMC11311425; doi:10.3390/foods13152358)
Supplement: Supplementary file 1 [file foods-13-02358-s001.zip › foods-3117619-supplementary.pdf]

Supplementary Materials

Table S1. Plackett & Burman experimental design with 20 trials (PB20) and 3 central points matrix.

	Coded matrix PB20													
**Treat**	**x1**	**x2**	**x3**	**x4**	**x5**	**x6**	**x7**	**x8**	**x9**	**x10**	**x11**	**x12**	**x13**	**x14**
1	1	-1	1	1	-1	-1	-1	-1	1	-1	1	-1	1	1
2	1	1	-1	1	1	-1	-1	-1	-1	1	-1	1	-1	1
3	-1	1	1	-1	1	1	-1	-1	-1	-1	1	-1	1	-1
4	-1	-1	1	1	-1	1	1	-1	-1	-1	-1	1	-1	1
5	1	-1	-1	1	1	-1	1	1	-1	-1	-1	-1	1	-1
6	1	1	-1	-1	1	1	-1	1	1	-1	-1	-1	-1	1
7	1	1	1	-1	-1	1	1	-1	1	1	-1	-1	-1	-1
8	1	1	1	1	-1	-1	1	1	-1	1	1	-1	-1	-1
9	-1	1	1	1	1	-1	-1	1	1	-1	1	1	-1	-1
10	1	-1	1	1	1	1	-1	-1	1	1	-1	1	1	-1
11	-1	1	-1	1	1	1	1	-1	-1	1	1	-1	1	1
12	1	-1	1	-1	1	1	1	1	-1	-1	1	1	-1	1
13	-1	1	-1	1	-1	1	1	1	1	-1	-1	1	1	-1
14	-1	-1	1	-1	1	-1	1	1	1	1	-1	-1	1	1
15	-1	-1	-1	1	-1	1	-1	1	1	1	1	-1	-1	1
16	-1	-1	-1	-1	1	-1	1	-1	1	1	1	1	-1	-1
17	1	-1	-1	-1	-1	1	-1	1	-1	1	1	1	1	-1
18	1	1	-1	-1	-1	-1	1	-1	1	-1	1	1	1	1
19	-1	1	1	-1	-1	-1	-1	1	-1	1	-1	1	1	1
20	-1	-1	-1	-1	-1	-1	-1	-1	-1	-1	-1	-1	-1	-1
21	0	0	0	0	0	0	0	0	0	0	0	0	0	0
22	0	0	0	0	0	0	0	0	0	0	0	0	0	0
23	0	0	0	0	0	0	0	0	0	0	0	0	0	0

Legend: x1 = β-Glucanase concentration (%w/w); x2 = β-Glucanase action time (min); x3 = Transglutaminase (TG) concentration (%w/w); x4 = temperature/time binomial (^{o}C/min) for TG; x5 = soy protein isolate–SPI (%w/w); x6 = oat flour-OF (%w/w); x7 = glutamine-Gt (%w/w); x8 = monosodium glutamate-MG(%w/w); x9 = acacia gum-AG(%w/w); x10 = cassava starch-CS (%w/w); x11 = coconut oil-CO (%w/w); x12 = soybean oil-SO (%w/w); x13 = sodium tripolyphosphate-ST (%w/w); x14 = β-Glucanase inactivation time (min).

Table S2. Aroma-active compounds identified in T6, T17 and T21 samples of fish fillet analogues and in stipe of *Pleurotus ostreatus* Hiratake mushrooms by HS-SPME-GC-MS analysis.

Class	Compounds	CAS	Treatment 6		Treatment 17		Treatment 21		Hiratake Stipe		Description odour	Tilapia fried in soybean oil[a]	
			Area %	IR	Area %	IR	Area %	IR	Area %	IR		IR	Description odour
Hydrocarbon	1-(Pent-1-ene-5-yl)-1,3-dimethyl-allene	2007-12-04	0.09	n.c.							n.f.		
	1,1,2-Trifluoro-2,5-bis(trifluoromethyl)hexane	1983-1355-0		1038	0.10	812			0.02	812	n.f.		
	1-Octene or Caprylene	111-66-0					1.59	792			Gasoline***		
	1-Pentene or Propylethylene	109-67-1		n.c.			0.24				Alkane* gasoline, Highly Disagreeable**		
	3-hexene or 2,3- dimethyl-	592-47-2	2.10	972							n.f.		
	Ethylbenzene	100-41-4			0,12	1061					Phenol, spice*; Aromatic, Pungent,Sweet, gasoline-like,**		
	cis-1,3-Difluoro-1,3-dimethylyclobutane	2398-09-06					0.01	867			n.f.		
	Nonane or n-Nonane	111-84-2							0.07	1100	Alkane*; Gasoline-like**		
	Cyclopropane or Trimethylene	75-19-4	0.26	n.c.							Mild, sweet, resembling solvent naphtha**		
	Methane, tetranitro or Tetranitromethane	509-14-8	3.20	n.c.	0.97	n.c.	2.62	n.c.			Pungent**		
	Pentane or n-Pentane	109-66-0	7.46	n.c.	2.61	n.c.	18.19	n.c.	0.13	n.c.	Alkane*		
	Propane, 2-nitro- or 2-Nitropropane	79-46-9	0.00	872			0.16	1003			Pleasant fruity**		
	Propene-1-D1	33922-80-4			0.03	1374					Faint, petroleum-like**		
	Propene-3-D1	1117-89-1			0.19	1004	0.07	1100			Faint, petroleum-like**		
	1,3-Octadiene	1002-33-1							0.08	825	n.f.		
	trans,trans-4,4,5,5-Tetramethyl-2,6-octadiene	75232-91-6	0.03	1307							n.f.		
Alcohols	(5Z)-Octa-1,5-dien-3-ol	50306-18-8			0.73	1375					Earthy, mushroom, gernaium, leafy, marine**		
	tert-Butanol-1-13C	75-65-0	0.10	1379							Camphor-like**		
	1,1-Dichloro-3-buten-2-ol	39638-32-9			0.04	1335			0.00		n.f.		
	1,2-Propanediol or Propylene glycolPropanediol	57-55-6					0.06		0.01		Practically odorless**		
	1-Butanol, 2-methyl- or 2-Methyl-1-butanol	137-32-6	0.43	n.c.							Cooked roasted with fruity or alcoholic undertones**		
	1-butanol,3-methyi-(impure) 2-Methyl-1-butanol	123-51-3	0.43	n.c.							n.f.		
	1-Heptanol	111-70-6			0.48	1355					Fragrant, Faint, Aromatic, Fatty**		
	1-Octen-3-ol or n- Oct-1-en-3-ol	3391-86-4			1.61	1378	1.34	1378			Mushroom*		
	-Hexanol or n-Hexanol	111-27-3							0.05	872	Sweet alcohol; pleasant, fatty, fruity, aromatic**		
	2-Heptanol or 2-Hydroxyhepneta	543-49-7									Mild alcohol, fresh lemon-like, grass-herbaceous**		
	2-Propanol or Isopropyl alcohol	67-63-0	0.35	n.c.			0.12	n.c.			Alcohol, pungent*		
	7-Octen-4-ol or 1-Octen-5-ol	53907-72-5							26.53	1381	Soybean**		
	3-Octanol or n-Octan-3-ol	589-98-0	1.79	1398	15.88	1397	27.66	1397			Mushroom*		
	1-Propanol, 2-methy or Isobutyl alcohol	78-83-1							0.10	n.c.	Alcohol, pungent*		
	1-Butanol, 3-methyl or 3-Methyl-1-butanol	123-51-3							1.72	n.c.	Whiskey, malt, burnt*		
	Ethanol or Ethyl alcohol	64-17-5	13.85	n.c.	2.59	n.c.	3.55	n.c.	4.74	n.c.	Vinous **		

	(-)-(3S,1'R)-3,4-Dihydro-7-nitro-3-(2-nitro-1-phenylethyl)-2(1H)-naphthalenone	40353-34-2	0.24	1295	0.08	1355					n.f.		
ketones	2,3-Butanedione or Diacetyl	0431-03-08			0.25	n.c.					Butter*	990	Creamy
	2-Butanone or Methyl ethyl ketone	78-93-3	0.29	n.c.							Ether*		
	2-Butanone, 3,3-dimethyl- or 3,3-Dimethyl-2-butanone	75-97-8			0.10	1206					Peppermint **		
	2-Pentanone, 3-methyl- or 3-Methyl-2-pentanone	565-61-7			0.09	n.c.					Mint *		
	2-Propanone or Acetone	67-64-1			0.23	890	0.33	890			Fruity**		
	3-Buten-2-one or Methyl vinyl ketone	78-94-4					0.04	1075			Mint *		
	3-Hepten-2-one or Hept-3-ene-2-one	1119-44-4			0.17						Pungent**		
	3-Octanone or EAK	106-68-3	1.54	1083	17.09	1083	9.73	1083	28.57	1084	Mild fruity **		
	Cyclobutanone, 2,3,3-trimethyl or 2,3,3-trimethylcyclobutanon	1449-49-6			0.15	792					n.f.		
	Acetylvaleryl	96-04-8							0.02	800	Buttery***		
	trans-8-Oxobicyclo[4.3.0]nonan-7-one	6051-03-02			0.03	1038					n.f.		
	2RS,3SR)-4-Acetoxy-2-methylbutane-1,3-diol 1-O-benzyl ethe	1216-84-8			0.08	1053					n.f.		
Esters	1-Chloroethyl acetate	0107-07-03			0.01	852					Pineapple*		
	2(3H)-Furanone, 5-methyl- or 5-methyl-2-oxo-2,3dihydrofuran	87598-08-01			0.04	818					Meat*		
	2-Ethenylcyclohex-2-enyl ethanoate	1205-42-1			0.18	894					Fruity**		
	Diphosphoric acid, diisooctyl ester or Di(iso-octyl)pyrophosphate	1506-38-3			1.9	872					n.f.		
	Formic acid, methyl ester or Methyl formate	107-31-3					0.01	n.c.			Agreeable **		
	Hept-enyl-2-acetate	16939-73-4	0.67	890							Fruit, sweet*		
	Isoamyl acetoacetate	2308-18-1	0.35	n.c.							Banana*		
	Nitrous acid, butyl ester or n-Butyl nitrite	544-16-1			0.15	845					Fruit*		
	Octanoic acid, ethyl ester or Ethyl caprylate	106-32-1			0.52	1297					Waxy ***		
	Propyl nitrite	543-67-9			0.01	854					Fruit*		
	Acetic acid, ethyl ester or Ethyl acetate	141-78-6							0.35	n.c.	Pineapple*		
	2-Butenal or Crotonaldehyde	4170-30-3	0.52	1016	0.77	n.c.					Flower* fruit*		
Aldehydes	2-Ethyl-2-hexenal	645-62-5			1.64	1007					Apple, green *		
	5-Hexenal	764-59-0	0.04	1004							Green, leaf *		
	Acetaldehyde or Ethanal	75-07-0	2.03	n.c.	1.03	866	0.24	n.c.			Pungent, ether *		
	Benzaldehyde or Phenylmethanal	100-52-7	1.01	962	22.56	961	0.4	962			Cocoa, almond *	1550	Almond
	Butanal, 2-methyl- or 2-Methylbutanal	96-17-3	17.55	n.c.	2.02	n.c.	6.72	n.c.			Cocoa, Fruity***		
	Butanal, 3-hydroxy or 3-Hydroxybutanal	107-89-1			1.45	915					n.f.		
	Butanal, 3-methyl- or 3-Methylbutanal	590-86-3	26.17	n.c.	4.73	n.c.	9.8	n.c.			Malt *		
	Hexanal or n-Hexanal	66-25-1	8.06	801	15.27	801					Grass, tallow, fat *	1090	Vegetative
	Octanal Oor n-Octanal	124-13-0	0	n.c.	0.25	1004					Fat, soap, lemon, green *	1297	Orangepeel
	Pentanal or n-Pentanal	110-62-3			1.18	n.c.					Almond, malt, pungent*		
	Propanal, 2-methyl or Isobutanal	78-84-2	3.66	n.c.	0.55	n.c.	0.55	n.c.			Pungent**	838	Malty
Acids carboxylics	Acetic acid or Ethylic acid	64-19-7	0.93	n.c.					0.23	n.c.	Sour		
	Butanoic acid, 3-methyl or Isovaleric acid	503-74-2					02.08	848			Sweat, acid, rancid		
	Quinoline-3-carboxylic acid	6480-68-8	0.03	977							n.f.		

	Methyl ester of 2S-(1,2,3,4,4a,5,6,7,8,8a.alpha.-Decahydro-1.alpha.,5.beta.-dihydroxy-44a.beta.-ethyl-8-oxonaphthalene-2.beta.-	67893-02-01	0.01	977	0.08	1066	0.14	956			n.f.		
	(S)-2-S - Butylfuran	4466-24-4					0.51	1038			Mild fruity**		
Furanos	2,3,5- Trimethylfuran	10504-04-08							0.14	834	Roasted meat*		
	2-Acetyl-5-methylfuran	1193-79-9							0.66	988	Strong, nutty, hay-coumarin odor**		
	Furan	110-00-9			0.26	1044					Ethereal **		
	Furan, 2-pentyl- or 2-Amylfuran	3777-69-3	1.26	989	1.12	989	1.89	989			Fruity***	1236	Fruity
	[9][3,6]-S-Tetrazinophane	14691-88-4	0.03		0.13		0.06				n.f.		
Other compounds	1,2,4,5-Tetrazine, 3,6-dipropyl- or 3,6-Di-2-pyridyl-s-tetrazine	1671-87-0			0.13	903					n.f.		
	2,3,4,4-Tretrapropyl-1-(trimethylsilyl)-1-(trimethylsilyloxy)-1,3-diaza-2,4-diborabutane		0.19	1396	0.08	1396	0.12	1396			n.f.		
	Acetonitrile or Methyl cyanide	75-05-8	0.03	792							Sweet, ethereal**		
	Cycloheptatrienylium, bromide or Tropylium bromide	5376-03-04			0.22	861					n.f.		
	Di-n-octyl	117-84-0					0.03	n.c.			Fruit*		
	Carbon dioxide	124-38-9							4.80	n.c.	Odorless **		
	6-Azabicyclo[3.2.1]octan-7-one	6142-56-9							0.02	n.c.	n.f.		
	Butane, 1-chloro-3-methyl or 1-Chloro-3-methylbutane	107-84-6							0.03	876	Black currant*		
	Cyclopropane, 1,1-dibromo-2-chloro-2-fluoro-	63708-08-07							1.00	n.c.	n.f.		
Total			34		47		28		21				

IR: Index Retention; CAS: Chemical Abstracts Service; D.O.: Description Odor; n.c.: not calculated; n.f.: not found; [a] [32] ;

* [67] Flavornet : https://www.flavornet.org/flavornet.html;

** [68] Pubchem:https://pubchem.ncbi.nlm.nih.gov/;

*** [69] The Good Scents Company: https://www.thegoodscentscompany.com/data/rw1155991.html

References

32. Liu, M.; Zhao, X.; Zhao, M.; Liu, X.; Pang, Y.; Zhang, M. Characterization of the Key Aroma Constituents in Fried Tilapia through the Sensorics Concept. Foods 2022, 11, 494. https://doi.org/10.3390/foods11040494.

67. FlavorNet: Database of Aroma Compounds. Available online: https://www.flavornet.org/flavornet.html (accessed on 29 May 2024).

68. PubChem: Open Chemistry Database. Available online: https://pubchem.ncbi.nlm.nih.gov/ (accessed on 29 May 2024).

69. The Good Scents Company. Good Scents Data: Aroma Chemical Information. Available online: https://www.thegoodscentscompany.com/data/ rw1155991.html (accessed on 29 May 2024).
